# Bank performance and Non-interest income diversification: Evidence from Chinese commercial banks

**DOI:** 10.1371/journal.pone.0321899

**Published:** 2025-05-19

**Authors:** Yuan Zhao, Yixi Mei, Jun Wang

**Affiliations:** 1 School of International Business, Southwestern University of Finance and Economics, Chengdu, China; 2 Fintech Innovation Center, Southwestern University of Finance and Economics, Chengdu, China; 3 School of Management Science and Engineering, Southwestern University of Finance and Economics, Chengdu, China; 4 The Financial Intelligence and Financial Engineering Key Lab of Sichuan Province, Southwestern University of Finance and Economics, Chengdu, China; An-Najah National University, PALESTINE, STATE OF

## Abstract

This paper aims to investigate the impact of non-interest income diversification on the performance of commercial banks in China. We conduct an empirical analysis using the panel data of 159 Chinese commercial banks from 2006 to 2020. The results indicate that there is a positive relationship between the degree of non-interest income diversification and bank performance. We suggest the commercial banks to actively develop diversified non-interest business and to balance the proportion of interest and non-interest income. Furthermore, we conduct heterogeneity analysis and find remarkable differences existing among different types of commercial banks. The results show that the effect of non-interest income diversification on bank performance is more significant for the small-sized or local commercial banks whose target customers are local residents. We also take a further analysis with interaction terms and find that the positive effect of non-interest income diversification on bank performance is stronger for those with higher market competitiveness. Our findings have implications for bank managers about expanding banks’ income resources through diversified business strategies and can provide experience for small or local banks seeking to advance the performance.

## 1. Introduction

Non-interest income (NII) is income generated by banks from sources other than interest payments [1]. There has been a substantial extension of off-balance sheet activities in numerous banks recently, and non-interest income is becoming an important source of bank revenue [2]. Traditionally, Chinese commercial banks rely on interest payments as their primary revenue source. However, banks’ net interest margins have been observed to be narrowing [3]. With the entry of overseas commercial banks, it is difficult for the domestic banks to differentiate themselves solely by their traditional interest business. Thus, Chinese commercial banks need to develop non-interest business in order to diversify their sources of income.

In 2001, the People’s Bank of China issued the Interim Provisions on Intermediary Business of Commercial Banks, which signals that the government has generally relaxed the restrictions on non-interest business, leading to a rapid increase in the proportion of non-interest income. From 2008 to 2020, the proportion of non-interest income of Chinese commercial banks increased substantially from 11.79% to 25.16% [4]. Chinese commercial banks have recognized the importance of non-interest income for improving competitiveness and spreading risks. Subsequently, numerous commercial banks have started to actively develop non-interest business. For instance, some state-owned commercial banks place particular emphasis on financial leasing, which is a significant component of non-interest income. Additionally, the joint-stock commercial bank such as Shanghai Pudong Development Bank provides one-on-one wealth management services for clients, so as to increase the non-interest income through charging asset management fees and commissions [5].

Challenges are faced by Chinese commercial banks with their rapid development. First, in comparison with the developed countries, Chinese commercial banks’ proportion of non-interest income is quite low [6]. Second, the internal structure of non-interest income of many Chinese commercial banks is strongly unbalanced. For instance, most commercial banks’ net income of poundage and commissions accounts for more than seventy percent of total non-interest income [7]. Thirdly, there is significant heterogeneity in the internal structure of non-interest income between large and small banks. Similar to the emerging economies in South Asian, large commercial banks in China tend to have a wider variety of non-interest business and their internal structure of non-interest income is more balanced than that of the small and medium-sized banks [8].

This study contributes to the literature by investigating the impact of non-interest income (NII) diversification on the performance of commercial banks. We have made an improvement on measuring the diversification level of non-interest income. We construct a new NII diversification index based on the Herfindahl-Hirschman index. Considering the inherent imbalance observed in the NII internal structure of Chinese commercial banks, we measure NII diversification by classifying the internal structure of NII as five segmentations, including net income of poundage and commissions, net income of investment, net income of exchange, net income of changes in fair value and other business income, in order to provide a more accurate measurement of NII diversification. Secondly, this study uses quantitative data analysis including a majority of Chinese commercial banks, whereas previous studies generally only focused on a few representative Chinese commercial banks. Besides, this study conducts heterogeneity analysis based on three dimensions: bank type, bank’s asset size and business scope. The results of these subsamples can provide insights for small or local banks in China.

The remainder of this paper is organized as follows. Section 2 reviews the literature and develops hypotheses. Section 3 introduces the variables and models. Section 4 presents the empirical results, robust tests and heterogeneity analysis, and gives a further analysis on the impact of market competitiveness. Section 5 concludes the paper and gives some implications.

## 2. Literature review and hypothesis development

The portfolio theory proposed by Markowitz [9] supports the significant effect of diversification process on business performance. The theory implies that banks with diversified portfolio can increase their profitability by minimizing the possibility of bank default [9]. Later on, a majority of studies argue that income diversification has a positive impact on bank performance. For instance, the results of Elsas et al. [10] show that improving the diversity of income can expand the sources of bank income, and thereby increasing the bank profitability. Similarly, Sanya and Wolfe [11] provide evidence that the increase in the diversification of income is conducive to improving bank profitability by carrying out a study on 226 commercial banks in 11 emerging economies. The theory of financial innovation also suggests a positive effect of income diversification on bank performance. On the one hand, banks can develop new financial products and services to attract more potential customer and expand revenue resources. On the other hand, the banks need to develop more diversified services such as non-interest business, in order to spread the risks of interest rate fluctuations [12]. Khraisha and Arthur [13] claims that the organizations could gain competitive advantages through developing innovative financial products or services. Meslier et al. [14] reach similar conclusions by providing evidence from an emerging economy. They confirm the positive effect of diversified non-interest business on bank profitability. Chen [15] conducts a panel data analysis of 24 large commercial banks in China and finds that increasing the proportion of non-interest income can improve the business performance of large commercial banks. On the basis of Chen ’s [15] study, Li [3] takes a further study based on a sample of 38 listed banks in China from 2011 to 2020. He also reports that the increased proportion of non-interest income and the diversified income structure can improve the stability and profitability of Chinese banks. These prior studies indicate that diversified non-interest business can benefit bank performance by diversifying revenue sources and decreasing reliance on traditional loan income. These prior studies indicate that diversified non-interest business can benefit bank performance by diversifying revenue sources and decreasing reliance on traditional loan income.

On the contrary, the agency theory seems to predict a negative impact of income diversification on financial performance [16]. According to Jensen [17], managers might undertake low-benefit or value-destroying diversification to expand the size of their business territories, for the purpose of managerial entrenchment which could benefit their personal positions. Studies provide evidence that banks’ reliance on non-interest activities may raise agency issue and moral hazard problems ([18,19]). Similarly, Stiroh and Rumble [20] find that increasing the proportion of non-interest income can increase the level of bank risk, because the bank employees lack experience in doing non-interest business. Later on, Berger et al. [21] claim that agency costs are higher than the benefits of income diversification for Chinese banks over the period of 1996–2006. Maudos [22] argues that banks in the European markets cannot achieve more stable income by increasing the proportion of non-interest income. The investment business and exchange business of non-interest income have high volatility, which might bring uncertainty and cause negative influence on bank performance. Kurniawan and Siswanto [23] examine the impact of non-interest income diversification on bank performance and conclude that diversification increases expenditure on regulatory activities and agency costs, and thereby decreasing bank performance. Lahouel et al. [24] analyze European commercial banks using a dynamic network model from the perspective of bank efficiency. Inconsistent with the previous studies, they find a nonlinear relationship between income diversification and bank stability. However, most of the studies mentioned above mainly focus on large state-owned banks or joint-stock banks that omitting the small or local banks.

Overall, there is no consensus on the effect of income diversification on bank performance. Empirical evidence from developed economies, especially from the U.S. market, indicating that income diversification reduces the profitability of U.S. banks (e.g. [25–27]). While most empirical studies on banks in developing countries indicate a positive relationship between income diversification and bank performance (e.g. [3,11,14,15,28–31]). Nevertheless, the theory of scope economy suggests that when the banks offer a range of services, the fixed costs can be apportioned through diversified business, leading to a reduction in total costs. A bank’s non-interest business can generate greater benefits with only a slight increase in marginal costs by leveraging its traditional balance sheet business [28]. Furthermore, according to the modern portfolio theory, business diversification can improve bank performance through mitigating the operational and systemic risk. Thus, we propose the hypothesis that the commercial banks’ non-interest income diversification has a positive effect on bank performance.

**H1:** the degree of a commercial bank’s non-interest income diversification has positive effect on bank performance.

Some experts propose that one of the key motivations behind diversification is the search for market competitive power [16,32]. By entering other markets through diversification, organizations are able to gain competitive power in the market and then improve their financial performance [16]. This is confirmed by Zhou and Liu [33] who find that enhancing banking competitiveness can lead to increased bank stability and liquidity, and thereby encouraging commercial banks to develop diversified non-interest business. Fang et al. [34] investigate a sample of Chinese banks over the period 2003–2017. They find that an increase in a bank’s competition can promote the profit efficiency and then improve the performance or profitability of the bank. In accordance with the implication of market-based view theory, we hypothesize that the positive effect of non-interest income diversification on bank performance is stronger for those with higher market competitiveness.

**H2:** the positive relationship between non-interest income diversification and bank performance is stronger for the commercial banks with higher market competitiveness.

## 3. Methodology

### 3.1. Data

This paper employs an unbalanced panel data set comprising 159 commercial banks in China from 2006 to 2020. We drop the period after the year of 2020 in order to eliminate the disordered impact of covid-19 pandemic. After excluding the extreme values, the final sample contains 2048 observations. In comparison with prior studies on Chinese banking sector, the sample of this paper is considerably broader. The subsamples consist of 6 state-owned commercial banks, 12 joint-stock commercial banks, 97 urban commercial banks and 44 rural commercial banks respectively. The data is mainly retrieved from Bloomberg and WIND database, with some missing data supplemented by the commercial banks’ annual reports. Bank recruitment data are sourced from the Bank Recruitment Website.

### 3.2. Variables

#### 3.2.1. Bank performance.

The dependent variable is bank performance. Following Chiorazzo et al. [35], Ghosh [36], Haw et al. [37], Hirtle et al. [38] and Quyen et al. [39], we use return on total assets (ROA) to measure the performance of commercial banks.

#### 3.2.2. Non-interest income diversification.

The independent variable is the non-interest income (NII) diversification of commercial banks. The sources of non-interest income include poundage and commission income, investment net income, exchange net income, fair value change net income, and other business income. Specifically, investment net income includes income from investments in stock or bond markets. Poundage and commission income includes income from payment and settlement services, credit card services and wealth management services. The fees generated by these services are an important part of the bank’s non-interest income. Other business income consists of financial advisory fees and asset management fees and compensation. Currently, there is no consensus definition for the NII diversification index in academia. We improve the measurement of NII diversification by creating a new NII diversification index based on the Herfindahl-Hirschman index. Our new index includes five sub-items: ratio of poundage and commission income (PR), ratio of investment net income to non-interest income (IR), ratio of exchange net income to non-interest income (ER), ratio of fair value change net income to non-interest income (FR), and ratio of other business income to non-interest income (OR*)*. The NII diversification index (DIVN) is then calculated by the following formula, which measures the balance of internal structure of non-interest income.


$$\begin{array}{c} DIVN=1-\left({PR}^{2}+{IR}^{2}+{ER}^{2}+{FR}^{2}+{OR}^{2}\right)\#(1) \end{array}$$ (1)

A higher value of DIVN indicates a more balanced internal structure of non-interest income, and consequently a higher degree of diversification. Conversely, a low value of DIVN suggests that non-interest income relies heavily on a few specific business areas.

#### 3.2.3. Control variables.

Following the prior studies [40–42], this paper takes some factors that are commonly agreed to have impact on bank performance as the control variables. The logarithm of total assets *(*lnAS) is used to measure the bank size. The non-performing loan ratio (NPL) is used to measure the security of credit assets. The cost income ratio (CIR) is used to measure the operational efficiency of commercial banks. The loan-to-asset ratio (LTA) is used to reflect the asset composition of banks [14,35]. Following Adesina [40] and Jiang et al. [43], the logarithm of provincial GDP (lnGDP) and provincial consumer price index (CPI) are used to reflect the macroeconomic factors. The specific variables and definitions are shown in Appendix A in S1 File.

### 3.3. Models

This study uses fixed effects regression for the panel data model. The model is constructed as follows:


$$\begin{array}{c} {ROA}_{it}=\beta_{0}+\beta_{1}{DIVN}_{it}+\beta_{2}{\ln AS}_{it}+\beta_{3}{NPL}_{it}+\beta_{4}{CIR}_{it}+\beta_{5}{LTA}_{it}+\beta_{6}{\ln GDP}_{it}+\beta_{7}{CPI}_{it}+T_{t}+\pi_{i}+\varepsilon_{it} \end{array}$$ (2)

where ${ROA}_{it}$ represents the operating performance of bank i at year t*.* ${DIVN}_{it}$ represents the degree of non-interest income diversification of bank i at year t. ${\ln AS}_{it}$, ${NPL}_{it}$, ${CIR}_{it}$,$\;{LTA}_{it}$, ${\ln GDP}_{it}$and ${CPI}_{it},$ are the control variables. $T_{t}$ is the year dummy variable. $\pi_{i}$ represents the unobserved individual impact of bank i. Both year and firm fixed effects are controlled. $\varepsilon_{it}$ is the standard error term.

## 4. Empirical results and discussion

### 4.1. Descriptive statistics

Table 1 reports the descriptive statistics of the variables. The median of non-interest income diversification (DIVN) surpasses its mean value, indicating a left-skewed distribution and suggesting a greater concentration of data to the right of the mean. Moreover, the average value of *DIVN* is only 0.049. It implies that the degrees of non-interest income diversification for Chinese commercial banks are relatively low. In addition, there is a notable disparity between the first and third quartiles of return on total assets (ROA), highlighting significant heterogeneity among the performance of Chinese commercial banks.

**Table 1:** Summary Statistics of the variables used in the analysis, 2006-2020.

variable	observations	mean	sd.	25%	50%	75%
$\begin{array}{c} ROA \end{array}$	2048	1.003	0.441	0.724	0.977	1.264
$\begin{array}{c} DIVN \end{array}$	2048	0.049	0.014	0.050	0.051	0.051
$\begin{array}{c} NPL \end{array}$	2048	1.719	1.392	0.950	1.460	1.950
$\begin{array}{c} CIR \end{array}$	2048	34.27	7.539	29.34	33.38	38.35
$\begin{array}{c} lnAS \end{array}$	2048	25.57	1.735	24.37	25.37	26.44
$\begin{array}{c} LTA \end{array}$	2048	48.5	9.993	41.93	49.43	56.21
$\begin{array}{c} lnGDP \end{array}$	2048	29.03	1.253	28.26	28.85	29.50
*CPI*	2048	102.6	1.581	101.7	102.3	103.1

Notes: The table reports summary statistics for the main variables used in the analysis.

### 4.2. Multicollinearity check

Table 2 shows the correlation matrix between the variables. The results indicate that the correlation coefficients between dependent and explanatory variables are not large enough to flag any collinearity problem. The model has not violated the perfect multicollinearity assumption. Besides, the Variance Inflation Factor (VIF) analysis reveals that the VIF values for each variable are less than 10, revealing no signs of multicollinearity between regressors.

**Table 2:** Correlation Matrix and VIF.

Variables	*ROA*	*DIVN*	*lnAS*	*NPL*	*CIR*	*LTA*	*lnGDP*	*CPI*	VIF
*ROA*	1.000								
*DIVN*	0.087	1.000							1.01
*lnAS*	-0.198	0.060	1.000						2.04
*NPL*	-0.415	-0.049	-0.123	1.000					1.11
*CIR*	-0.408	-0.026	-0.156	0.200	1.000				1.07
*LTA*	0.095	-0.032	-0.204	0.196	0.086	1.000			1.26
*lnGDP*	-0.077	0.045	0.640	-0.123	-0.058	0.140	1.000		1.99
*CPI*	0.129	0.025	-0.124	0.032	-0.036	0.082	-0.087	1.000	1.03

Notes: The table reports the correlation matrix between the variables used in the analysis. VIF is reported in the right column in the table.

### 4.3. Unit root test

In order to identify potential associations between variables, the research employs panel unit root tests on the data. Specifically, both the ADF-Fisher and Phillips-Perron for unit root tests are applied to enhance the robustness of the results. Table 3 shows that all series exhibit a stationary level of integration I (0), thereby rejecting the null hypothesis of non-stationarity.

**Table 3:** Panel unit root tests.

Variables	ADF-Fisher Chi-square	Phillips-Perron Chi-square	Results
*ROA*	425.45^***^	393.03^***^	I(0)
*DIVN*	964.50^***^	1635.4^***^	I(0)
*lnAS*	811.76^***^	1665.3^***^	I(0)
*NPL*	819.26^***^	1226.0^***^	I(0)
*CIR*	433.30^***^	702.05^***^	I(0)
*LTA*	504.87^***^	472.47^***^	I(0)
*lnGDP*	913.21^***^	2247.4^***^	I(0)
*CPI*	373.85^**^	1962.3^***^	I(0)

Notes: The table reports the panel unit root tests on the data.

*** p < 0.01, ** p < 0.05, * p < 0.1.

### 4.4. Lagrange multiplier tests

Following Abdullah’s [44] study, we use the Breusch-Pagan and White approaches to perform Lagrange multiplier test. The results of the proper model selection tests are shown in Table 4. The p-values for both tests are notably smaller than any conventional significance level, indicating a rejection of the null hypothesis and the presence of heteroskedasticity in the data.

**Table 4:** Lagrange multiplier tests.

Test	$\chi^{2}\;$Stat.	Prob.
Breusch-Pagan	2458.4	0.0000
White	274.53	0.0000

### 4.5. Baseline results

The regression results of the ordinary least squares estimation (OLS) and the two-way fixed effects model (FE) are shown in Table 5, with standard errors are clustered on the bank level.

**Table 5:** OLS and FE estimate of non-interest income diversification and bank performance.

	(i) OLS	(ii) FE
$\begin{array}{c} DIVN \end{array}$	1.906***	0.935**
	(0.476)	(0.413)
$\begin{array}{c} lnAS \end{array}$	-0.146***	-0.108***
	(0.010)	(0.024)
$\begin{array}{c} NPL \end{array}$	-0.132***	-0.096***
	(0.005)	(0.005)
$\begin{array}{c} CIR \end{array}$	-0.023***	-0.022***
	(0.001)	(0.001)
$\begin{array}{c} LTA \end{array}$	0.001	0.004***
	(0.001)	(0.001)
$\begin{array}{c} lnGDP \end{array}$	0.009	-0.020
	(0.016)	(0.057)
$\begin{array}{c} CPI \end{array}$	0.016***	-0.016
	(0.004)	(0.013)
$\begin{array}{c} Constant \end{array}$	3.668***	6.536***
	(0.565)	(2.206)
Observations	2,048	2,048
Adjusted R-squared	0.482	0.608
Year fixed effect		YES
Firm fixed effect		YES

Notes: The robust standard error is reported below the coefficient estimates in brackets.

*** p < 0.01, ** p < 0.05, * p < 0.1.

In Table 5, the results of OLS (column i) and FE estimations (column ii) show that the diversification of non-interest income has a significant and positive impact on bank performance. In Table 5, we can see that one unit increase in bank non-interest income diversification leads to an increase in bank performance by 1.906 and 0.935 respectively. The results support our hypothesis H1. While our findings are not in line with the prediction of agency theory about the negative effect of income diversification on bank performance. Therefore, the results are inconsistent with Berger et al.’s [21] argument that the agency costs will overcome the benefits of developing non-interest business. Our results also contrast those of Stiroh and Rumble [20] and Lahouel et al. [24] who study the commercial banks in the U.S. and European economies. One possible reason is that, 0n October 31 2006, Chinese government has amended the *Banking Supervision Law of the People’s Republic of China*, which strengthened the supervision and regulation over banking industry. Since then, Chinese banks have adopted more strict supervision mechanism [45]. As the bank managers are under strict supervision, the agency problem in Chinese banks may not as severe as that of the developed countries since 2007. It is evident that although the main driving forces behind the income diversification of banks in the developing countries may be similar to those in the developed countries, the differences in the institutional arrangement in these markets possibly lead to different diversification effects [30].

Another possible explanation is that the diversification of non-interest income can help Chinese commercial banks decrease their potential risks. In comparison with over-relying on a single non-interest business, diversified non-interest business can spread risks to some extent, especially for those with high-volatility. Furthermore, carrying out diversified non-interest business enables Chinese commercial banks to attract more potential customers and increase their competitiveness, thereby improving their financial performance.

Our results support the implications of modern portfolio theory, theory of financial innovation and the theory of scope economy. It is consistent with the prior study of Addai et al. [31] who investigate the African banks’ income diversification and performance during 2011–2018. They find that, with limited capital in terms of technology and human resource in African banks, the income diversification in developing markets may give different perspectives than in developed markets. Other empirical evidence from developing countries such as Meslier et al. [14], Chen [15], Nguyen [28], Ahamed [29], and Brahmana et al. [30], also suggest that income diversification improves bank performance. Our study supports the findings of these previous studies in developing countries.

In addition, the results in Table 5 reveal that bank size (lnAS) is negatively related to bank performance, implying that expanding bank size is not conducive to improving bank performance. It is because that with the expansion of bank scale, the management costs rise as well. The coefficients on non-performing loan ratio (NPL) are significantly negative, indicating that an increase of non-performing loan ratio will decrease bank performance. An excessive proportion of non-performing loans reduces the asset quality of banks, which requires banks to bear greater loan default risk, and banks also need more profits to offset the possible losses caused by non-performing loans. The cost income ratio (CIR) is also negatively related to bank performance. The higher the cost income ratio, the weaker banks’ capital utilization ability, and the harder to generate more revenue for the banks. This is in line with the empirical results of Pasiouras and Kosmidou [46]. The loan-to-asset ratio (LTA) is positively associated with bank performance, consistent with the findings of Ahamed [29].

### 4.6. 2SLS and system GMM estimations

This paper also uses two-stage least squares (2SLS) and two-step system generalized moment estimation (GMM) to control for the interference of endogenous problems, as shown in Table 6. For the selection of instrumental variable, considering that the bank’s diversified non-interest business requires the support of related employees, the more employees working in the positions of non-interest income, the more likely they can support the non-interest business. Therefore, the ratio of employees in non-interest income positions (Recruit) is used as an instrumental variable (measured as the number of non-interest income related jobs recruited divided by the total number of jobs recruited). The data are obtained from the China Bank Recruitment Website, which summarizes the recruitment information of commercial banks in China. The information on the recruitment of non-interest-income related positions can be obtained through keyword determination.

**Table 6:** 2SLS and two-step system GMM estimation.

	(i) First stage of 2SLS	(ii) Second stage of 2SLS	(iii) System GMM
Recruit	0.024***		
	(0.006)		
DIVN		11.231**	5.055**
		(5.503)	(2.493)
Control	YES	YES	YES
Observations	2,048	2,048	2,048
F statistic	11.20	45.27	
Adjusted R-squared	0.018	0.333	
Year fixed effect	YES	YES	
Firm fixed effect	YES	YES	
AR(2)			0.23
Hansen			0.78

Notes: The robust standard error is reported below the coefficient estimates in brackets.

*** p < 0.01, ** p < 0.05, * p < 0.1.

In column (i) and (ii) in Table 6, the F-statistics of 2SLS are greater than the critical value of weak instrumental variables, indicating that there is no weak instrumental variables problem. Similarly, in column (iii) in Table 6, the results of the Hansen test and ARCH test of two-step system GMM estimation are both above 0.1, confirming the validity of the instrumental variables and ruling out autocorrelation issues. The standard errors are calculated using the finite sample correction in Windmeijer [47]. In the first stage of 2SLS, the results indicate a significant positive correlation between the instrumental variables and the explanatory variables, aligning with our expectations. The results in column (iii) in Table 6 also show that the coefficients on DIVN are significant and positive. By utilizing instrumental variables to address potential endogeneity issues, the effect of non-interest income on bank performance remains consistent, and the overall model fit remains consistent.

### 4.7. Robustness test

#### 4.7.1. Replacing the measurement of key variable.

In this section, we examine whether our primary results are robust by applying an alternative measure of core explanatory variable. Previous studies use different methods to define and measure non-interest income segmentation. Following Li [3], this paper adopts an alternative method of measuring non-interest income diversification ($DIVN_{new}$). The formula for calculating the new index is as follows:


$$\begin{array}{c} DIVN_{new}=1-\left({PR}^{2}+{IR}^{2}+{OR_{new}}^{2}\right)\#(3) \end{array}$$ (3)

$$\begin{array}{c} OR_{new}=ER+IR+OR\#(4) \end{array}$$ (4)

where $DIVN_{new}$ represents an alternative measure for non-interest income diversification. PR represents the ratio of poundage to non-interest income. IR is the ratio of investment net income to non-interest income. $OR_{new}$ is the proportion of other non-interest income, which is equal to the sum of ratio of exchange net income to non-interest income (ER), ratio of net income from changes in fair value to non-interest income (FR), and ratio of other business income to non-interest incomeOR). And then we get the adjusted benchmark regression model as follows:


$$\begin{array}{c} {ROA}_{it}=\beta_{0}+\beta_{1}{DIVN_{new}}_{it}+\beta_{2}{\ln AS}_{it}+\beta_{3}{NPL}_{it}+\beta_{4}{CIR}_{it}+\beta_{5}{LTA}_{it}+\beta_{6}{\ln GDP}_{it}+\beta_{7}{CPI}_{it}+T_{t}+\pi_{i}+\varepsilon_{it} \end{array}$$ (5)

To test the robustness of the results, we employ the ordinary least squares estimation (OLS), the two-way fixed effect model and two-step system generalized moment estimation (System GMM). The standard errors are clustered on the bank-level, and the results of Hansen test and ARCH test confirm the validity of model. Table 7 reports the regression results of the adjusted regression model. Consistent with the results of the basic model (Equation 2), the coefficients on non-interest income diversification ($DIVN_{new}$) in the adjusted model (Equation 5) are also significantly positive, which proves robustness of the empirical results.

**Table 7:** Robust test.

	(i) OLS	(ii) FE	(iii) System GMM
$DIVN_{new}$	0.572***	0.290***	1.435***
	(0.075)	(0.100)	(0.389)
$\begin{array}{c} lnAS \end{array}$	-0.142***	-0.109***	-0.210***
	(0.010)	(0.040)	(0.028)
$\begin{array}{c} NPL \end{array}$	-0.127***	-0.094***	-0.190***
	(0.005)	(0.012)	(0.023)
$\begin{array}{c} CIR \end{array}$	-0.022***	-0.021***	-0.030***
	(0.001)	(0.002)	(0.003)
$\begin{array}{c} LTA \end{array}$	-0.000	0.003*	-0.000
	(0.001)	(0.002)	(0.002)
$\begin{array}{c} lnGDP \end{array}$	0.012	-0.038	0.036
	(0.016)	(0.078)	(0.039)
$\begin{array}{c} CPI \end{array}$	0.015***	-0.015	0.013**
	(0.004)	(0.019)	(0.005)
Constant	3.779***	7.022**	5.439***
	(0.561)	(3.000)	(1.045)
Observations	2,048	2,048	2,048
Adjusted R-squared	0.491	0.611	
Year fixed effect		YES	
Firm fixed effect		YES	
AR(2) p-value			0.19
Hansen p-value			0.80

Notes: The robust standard error is reported below the coefficient estimates in brackets.

*** p < 0.01, ** p < 0.05, * p < 0.1.

#### 4.7.2. Test for linearity.

As some experts argue that there is a nonlinear relationship between non-interest income and financial stability of European commercial banks (e.g. [24,48,49]), it is essential for us to consider including nonlinear terms in the model to examine potential diminishing returns. Thus, we add quadratic (*DIVN2*) and cubic (*DIVN3*) terms of non-interest income diversification (*DIVN*) in the model, and the FE results are shown in column (i) and column (ii) in Table 8. It shows that the coefficients on *DIVN2* and *DIVN3* are not statistically significant, indicating that the nonlinear relationship between non-interest income and performance does not exist.

**Table 8:** FE estimate of nonlinear regression model.

	(i) *Model with DIVN2*	(ii) *Model with DIVN3*
*DIVN*	0.575	0.026
	(1.201)	(1.386)
*DIVN2*	-0.242	-0.012
	(-1.491)	(-0.851)
*DIVN3*		-0.003
		(-1.194)
*lnAS*	-0.110***	-0.109***
	(-4.484)	(-4.459)
*NPL*	-0.096***	-0.095***
	(-18.080)	(-17.695)
*CIR*	-0.022***	-0.021***
	(-20.769)	(-20.384)
*LTA*	0.004***	0.003***
	(3.585)	(3.139)
*CPI*	-0.016	-0.015
	(-1.215)	(-1.122)
*lnGDP*	-0.015	-0.034
	(-0.260)	(-0.590)
*Constant*	6.636***	7.019***
	(2.929)	(3.096)
Observations	2,048	2,048
R-squared	0.732	0.733
Company FE	YES	YES
Year FE	YES	YES

Notes: The t-statistics is reported below the coefficient estimates in brackets.

*DIVN2* and *DIVN3* is the quadratic and cubic term of *DIVN* respectively.

*** p < 0.01, ** p < 0.05, * p < 0.1.

Additionally, we also take the likelihood ratio (LR) test to examine whether the nonlinear models outperform the linear model. In Table 9, the test results show that the nonlinear models do not have a better fit than our original linear model. Thus, there is a linear link between the non-interest income diversification and performance of Chinese commercial banks.

**Table 9:** LR test.

	*LR* $\chi^{2}$	*P-value*
Linear model *versus* nonlinear model with a quadratic term	2.44	0.119
Linear model *versus* nonlinear model with a cubic term	5.83	0.055

### 4.8. Heterogeneity analysis

This section conducts a heterogeneity analysis of banks’ non-interest income diversification according to the commercial banks’ asset size, business scope and ownership structure.

#### 4.8.1. Commercial banks with different sizes.

Generally, bank size can be distinguished according to their asset size or business scope. Following the studies of Hirtle et al. [38], and Tommaso and Thornton [50], the full sample is divided into two subsamples according to the commercial banks’ median asset sizes. The results are shown in column (i) and (ii) in Table 10. We also divide the full sample into two subsamples according to their business scope. The local commercial banks specializing in regional operations whose business scope is much narrower than that of the national commercial banks. The results of national and local commercial banks are shown in column (iii) and (iv) in Table 10.

**Table 10:** Heterogeneity analysis of banks with different sizes.

	Asset size		Business scope	
	(i) Large banks	(ii) Small banks	(iii) National banks	(iv) Local banks
$\begin{array}{c} DIVN \end{array}$	0.175	0.492***	0.637	0.293**
	(0.139)	(0.053)	(1.366)	(0.130)
$\begin{array}{c} lnAS \end{array}$	-0.138**	-0.137**	0.095	-0.130***
	(0.066)	(0.066)	(0.076)	(0.044)
$\begin{array}{c} NPL \end{array}$	-0.072***	-0.129***	-0.033*	-0.108***
	(0.016)	(0.016)	(0.016)	(0.013)
$\begin{array}{c} CIR \end{array}$	-0.022***	-0.019***	-0.011***	-0.022***
	(0.003)	(0.002)	(0.003)	(0.002)
$\begin{array}{c} LTA \end{array}$	0.002	0.003	0.006*	0.003*
	(0.002)	(0.002)	(0.003)	(0.002)
$\begin{array}{c} lnGDP \end{array}$	0.022	-0.113	-0.338**	-0.064
	(0.094)	(0.140)	(0.118)	(0.083)
$\begin{array}{c} CPI \end{array}$	-0.030	0.007	0.219***	-0.015
	(0.025)	(0.026)	(0.058)	(0.020)
$\begin{array}{c} Constant \end{array}$	7.650**	7.507	-13.501**	8.273***
	(3.814)	(4.954)	(4.997)	(3.087)
Year fixed effect	YES	YES	YES	YES
Firm fixed effect	YES	YES	YES	YES
Observations	1,027	1,021	264	1,784
Adjusted R-squared	0.589	0.647	0.677	0.623

Notes: The robust standard error is reported below the coefficient estimates in brackets.

*** p < 0.01, ** p < 0.05, * p < 0.1.

The results show that non-interest income diversification *(DIVN*) is significantly and positively related with the performance of small-sized commercial banks. However, the diversification of non-interest income has no significant impact on large commercial banks’ performance. The findings support the previous studies of Ahamed [29], Jiang [43] and O’cass and Ngo [51]. One possible explanation is that, the non-interest businesses of small banks have developed rapidly in recent years, while the large banks’ income sources still mainly depend on interest-income rather than non-interest income. Another possible explanation is that there is scale economy effect in the banking industry [3,52,53]. Zhou and Li [54] argue that, in comparison with large banks, the relatively smaller or medium-sized banks have more obvious scale economy effect. The economies of scale that brought by the diversified business will cause an important impact on the performance and behavior of smaller-sized banks. Relevant studies include Humphrey [53], Lawrence [55], Benston [56], and our results are consistent with these prior studies.

In column (iv) in Table 10, we can see that the local banks’ non-interest income diversification has a significant and positive effect on their bank performance. In comparison, the coefficient on the non-interest income diversification *(DIVN)* of national banks is insignificant as shown in column (iii) in Table 10. This is consistent with the findings of Mercieca et al. [57]. They argue that it is because the local banks can leverage their focus on selected market segments to provide differentiated services, and then to gain a competitive advantage on non-interest business. We also give some possible explanations for the results. First, national banks and local banks target at different customer groups. The local banks tend to focus on local customers and carry out more targeted business which are more closely linked with local customers’ demand. While the national banks are primarily located in large cities, and their business tend to focus on the universal applicability across different regions. Second, the costs of diversifying non-interest business are much larger for the national banks in comparison with the local banks. Therefore, the national banks prefer to carry out business related to interest income rather than non-interest income.

#### 4.8.2. Commercial banks of different ownership structure.

In this section, the commercial banks are categorized by their ownership structure, including state-owned, joint-stock, urban and rural commercial banks. State-owned commercial banks refer to the banks that are directly invested and controlled by the state. Joint-stock commercial banks are owned by multiple shareholders. Urban and rural commercial banks are funded by local governments or enterprises to serve local businesses and residents, while rural commercial banks are primarily serving rural areas. Table 11 shows the regression results of the two-way fixed effects models of the four subsamples. In column (i) in Table 11, the coefficient on the non-interest income diversification *(DIVN)* of state-owned banks is insignificant, and its magnitude is very small. It indicates that the non-interest income diversification is insignificant for the performance of state-owned banks. The results in Table 11 are consistent with the results in Table 10, as the state-owned banks are also large banks in China.

**Table 11:** Heterogeneity analysis of commercial banks of different ownership structure.

	(i) State-owned banks	(ii) Joint-stock banks	(iii) Urban banks	(iv) Rural banks
$\begin{array}{c} DIVN \end{array}$	0.053	0.520	0.133**	0.529***
	(0.036)	(1.383)	(0.063)	(0.068)
$\begin{array}{c} lnAS \end{array}$	0.051	0.064	-0.127***	-0.109
	(0.192)	(0.090)	(0.048)	(0.165)
$\begin{array}{c} NPL \end{array}$	-0.063***	-0.024	-0.092***	-0.125***
	(0.015)	(0.023)	(0.017)	(0.016)
$\begin{array}{c} CIR \end{array}$	-0.002	-0.015***	-0.023***	-0.019***
	(0.008)	(0.003)	(0.002)	(0.005)
$\begin{array}{c} LTA \end{array}$	0.009	0.009**	0.003	0.004
	(0.008)	(0.004)	(0.002)	(0.004)
$\begin{array}{c} lnGDP \end{array}$	-0.237	-0.366**	0.017	-0.373
	(0.224)	(0.163)	(0.092)	(0.224)
$\begin{array}{c} CPI \end{array}$	0.133**	0.260***	-0.031	0.071
	(0.040)	(0.079)	(0.023)	(0.052)
$\begin{array}{c} Constant \end{array}$	-7.054	-16.111**	7.465**	7.716
	(5.514)	(6.679)	(3.336)	(9.872)
Year fixed effect	YES	YES	YES	YES
Firm fixed effect	YES	YES	YES	YES
Observations	86	178	1,257	527
Adjusted R-squared	0.795	0.696	0.627	0.646

Notes: The robust standard error is reported below the coefficient estimates in brackets.

*** p < 0.01, ** p < 0.05, * p < 0.1.

In column (iii) and (iv) in Table 11, the results show that the non-interest income diversification has a significantly positive effect on the performance of urban and rural banks. The results support the study of Wang and Zhao [58] who argue that the rural commercial banks can obtain higher marginal returns from the diversified non-interest business than that of the state-owned commercial banks in China. We can see that the results in column (iii) and (iv) in Table 11 are also consistent with the results in column (iv) in Table 10, as both urban and rural commercial banks are belonging to local commercial banks.

### 4.9. Further analysis: the impact of market competitiveness on the relationship between non-interest income diversification and bank performance

In this section, we further explore whether a commercial bank’s market competitiveness has an impact on the relationship between non-interest income diversification and its performance. Some prior studies point out that improving banking competitiveness promotes the diversification of income structure, and thereby increasing bank profitability [34]. Following Angelini and Cetorelli [59], the Lerner index is introduced to measure the level of market competitiveness of commercial banks. The Lerner index formula is shown below.


$${Lerner}_{it}\;=\;\frac{P_{it}-{MC}_{it}}{P_{it}}$$ (6)

where $P_{it}$ represents the average output price of bank *i* at year *t*, expressed as the ratio of operating income *t*o total income; ${MC}_{it}$ represents the marginal cost of bank *i* at year *t*. Then we can get the Lerner index of commercial banks, ranging between 0 and 1. The larger the Lerner index, the higher the level of a commercial bank’s market compe*t*itiveness.

We introduce the interaction term of market competitiveness and diversification of non-interest income into the benchmark regression model (Equation 2), and then we get the following model (Equation 7).


$$\begin{array}{cc} {ROA}_{it} & =\beta_{0}+\beta_{1}{DIVN}_{it}+\beta_{2}{Lerner\times DIVN}_{it}+\beta_{3}{Lerner}_{it}+\beta_{4}{\ln AS}_{it}\\ & +\beta_{5}{NPL}_{it}+\beta_{6}{CIR}_{it}+\beta_{7}{LTA}_{it}+{\beta_{8}\ln GDP}_{it}+\beta_{9}{CPI}_{it}+\varepsilon_{it} \end{array}$$ (17)

where $Lerner\times {DIVN}_{it}$ is the interaction term of market competitiveness and non-interest income diversification. The two-step System GMM estimation of Equation 7 is shown in column (i) in Table 12. Moreover, we employ another measurement of banking competitiveness which is the Boone index [60] as a robust test and present the results in column (ii) in Table 12. The robustness is aligned with the results in column (i) in Table 12.

**Table 12:** The effect of market competitiveness on the relationship between non-interest income diversification and bank performance.

	(i) (ii) *Two-step System GMM Robustness*	
$\begin{array}{c} DIVN \end{array}$	0.171***	0.209***
	(0.051)	(0.077)
$\begin{array}{c} Lerner\text{ × }DIVN \end{array}$	0.021**	
	(0.010)	
$\begin{array}{c} Lerner \end{array}$	0.077***	
	(0.014)	
$\begin{array}{c} Boone\text{ × }DIVN \end{array}$		0.316**
		(0.143)
$\begin{array}{c} Boone \end{array}$		0.470***
		(0.181)
$\begin{array}{c} lnAS \end{array}$	-0.147***	-0.179***
	(0.028)	(0.035)
$\begin{array}{c} NPL \end{array}$	-0.142***	-0.198***
	(0.021)	(0.022)
$\begin{array}{c} CIR \end{array}$	-0.028***	-0.032***
	(0.003)	(0.003)
$\begin{array}{c} LTA \end{array}$	-0.008***	-0.002
	(0.002)	(0.002)
$\begin{array}{c} lnGDP \end{array}$	0.036	-0.022
	(0.031)	(0.041)
$\begin{array}{c} CPI \end{array}$	0.014***	0.018***
	(0.004)	(0.005)
$\begin{array}{c} Constant \end{array}$	4.021***	6.035***
	(0.888)	(0.967)
Observations	1,953	1,950

Notes: The robust standard error is reported below the coefficient estimates in brackets.

*** p < 0.01, ** p < 0.05, * p < 0.1.

Table 12 shows that the coefficients on the interaction terms (Lerner×DIVN and Boone×DIVN) are significantly positive, confirming our conjecture that an increase in a bank’s market competitiveness promotes the positive impact of non-interest income diversification on bank performance. Moreover, the coefficient on Lerner is significant and positive, indicating that banks with stronger market competitiveness can have better performance. It is consistent with the study of Algeri et al [61], who point out that the historical monopoly power of large commercial banks has been diminished by the constant growth of market competition, which has also enhanced the markets for fair competition. Our findings imply that banks can improve their performance through articulating their competitive advantages to attract more potential customers and to occupy additional markets. In particular, they can develop a variety of non-interest businesses that are more individualized and tailored to their target markets, in order to differentiate themselves from their competitors.

## 5. Conclusion, implications and limitations

### 5.1. Conclusion

This study proves that the degree of non-interest income diversification has a significant and positive effect on the performance of commercial banks. The improvement we made on measuring the degree of a bank’s non-interest income diversification might be helpful for the future studies. Our findings indicate that commercial banks that engage in various diversified non-interest business can improve their performance, which aligns with the study of Li [3], Sanya and Wolfe [11], Meslier et al. [14] and Chen [15] on emerging economies. In order to ensure the robustness of our results, we conduct robust test, endogeneity test and heterogeneity analysis. The heterogeneity analysis reveals that, in comparison with large or national commercial banks, the non-interest income diversification of small or local commercial banks has a greater impact on bank performance. We also find that the degree of non-interest income diversification has a more significant promoting effect on bank performance for the commercial banks with stronger market competitiveness.

### 5.2. Implications

The study has several important implications. First, it adds signiﬁcant evidence to the existing literature that diversifying non-interest income can improve financial performance of Chinese commercial banks. The aforementioned findings have practical implications for policy makers and bank managers seeking to advance the performance of commercial bank. With the increased competition in banking industry, diversifying the businesses related to non-interest income is important for Chinese commercial banks. Furthermore, we provide evidence that banks with different asset sizes or business scopes have different diversification effects on bank performance. Thus, we suggest the small or local commercial banks to develop non-interest income-related financial services and products tailored to satisfy the demands of local clients, in order to gain comparative advantages. They can also fully utilize the benefits of diversification to expand income resources.

### 5.3. Limitations and future research

This study has some limitations that should be stressed. First, for the control variables, we use provincial GDP and provincial CPI to reflect the macroeconomic factors, while some other macro-economic factors may have been omitted in this study. Further discussion and investigation are required on analyzing the influence of macro-economic factors on income diversification and bank performance. Second, this study specifically focuses on the commercial banks in China. While China’s unique regulatory environment may limit the generalizability of the results in other economies. Thus, in our future research, we will deploy the algorithm used in the study of Agrrawal and Clark [62] to produce rankings of interest income versus non-interest income banks, in order to apply the findings to banks in different markets. Moreover, the sample period of this study is from 2006 to 2020 that excluding the time period of covid-19 pandemic, we will conduct a comparative study of bank performance before and after the pandemic in our near future research. Additionally, the artificial intelligence (AI) is becoming increasingly important that plays an important role in helping bank managers identify potential risks and improve operational efficiency [63]. Further work will be extended to the impact of AI on income structure and bank performance.

## Supporting information

S1 File (DOCX)
