# Supplementary material for: Bank performance and Non-interest income diversification: Evidence from Chinese commercial banks
Source: PLoS One. 2025 May 19;20(5):e0321899. doi: 10.1371/journal.pone.0321899 (PMC12088593; doi:10.1371/journal.pone.0321899)
Supplement: S1 File — (DOCX) [file pone.0321899.s001.docx]

**Appendix A.**

|  | Variable name | Symbol | Definition |
| --- | --- | --- | --- |
| Dependent variable | Return on total assets | $\text{ROA}$ | Net profit / total assets |
| Independent variable | Diversification of non-interest income | $\text{DIVN}$ | 1 - ( ${PR}^{2}$ + ${IR}^{2}$ + ${ER}^{2}$ + ${FR}^{2}$ + ${OR}^{2}$ ) |
|  | An alternative measure for non-interest income diversification | $DIVN_{new}$ | $1-\left( {PR}^{2}+{IR}^{2}+{OR_{new}}^{2} \right)$ |
| Control variables | Bank size | $\ln\text{AS}$ | Logarithm of total assets |
|  | Non-performing loan ratio | $\text{NPL}$ | NPL balance / total loan balance |
|  | Cost income ratio | $\text{CIR}$ | Business and management expenses / operating income |
|  | Loan-to-asset ratio | $\text{LTA}$ | Total loans / total assets |
|  | Macroeconomic development level | $\ln\text{GDP}$ | Logarithm of provincial GDP |
|  | Price level | *CPI* | Provincial Consumer Price Index |
| Items used to calculate *DIVN* | Ratio of poundage | *PR* | Ratio of poundage to non-interest income |
|  | Ratio of investment net income | *IR* | Ratio of investment net income to non-interest income |
|  | Ratio of exchange net income | *ER* | Ratio of exchange net income to non-interest income |
|  | Ratio of fair value change net income | *FR* | Ratio of fair value change net income to non-interest income |
|  | Ratio of other business income | *OR* | Ratio of other business income to non-interest income |
|  | An alternative measure for ratio of other business income | $OR_{new}$ | $ER+IR+OR$ |

**Table A1. Variables definitions**
